# Fulminant bilateral blindness despite initially normal neuroimaging in rhinocerebral mucormycosis

**DOI:** 10.1016/j.idcr.2026.e02772

**Published:** 2026-09-15

**Authors:** Nur Beyza Tukek Kilicaslan, Muhammed Enes Kardan, Hulya Duran

**Affiliations:** a Clinic of Internal Medicine, Tekirdag Dr Ismail Fehmi Cumalioglu City Hospital, Tekirdag, Turkey; b Clinic of Infectious Diseases and Clinical Microbiology, Tekirdag Dr Ismail Fehmi Cumalioglu City Hospital, Tekirdag, Turkey; c Department of Medical Microbiology, Tekirdag Dr Ismail Fehmi Cumalioglu City Hospital, Tekirdag, Turkey

**Keywords:** Bilateral blindness, Diabetic ketoacidosis, Orbital apex syndrome, Rhinocerebral mucormycosis

## Abstract

**Background:**

Rhinocerebral mucormycosis is a rapidly progressive and frequently fatal invasive fungal infection that most commonly occurs in patients with uncontrolled diabetes mellitus in the setting of diabetic ketoacidosis.

**Case presentation:**

We present a woman in her 70 s with diabetic ketoacidosis, who developed right-sided facial numbness and ptosis, followed by acute, complete bilateral vision loss within hours. Initial cranial CT and diffusion MRI were unremarkable despite rapidly evolving cranial neuropathies. This created significant diagnostic uncertainty and prompted consideration of alternative diagnoses, including giant cell arteritis. Nevertheless, markedly elevated inflammatory markers and rapidly worsening ophthalmoplegia prompted further investigation. Orbital MRI and paranasal sinus CT revealed postseptal orbital involvement and extensive pansinusitis. Nasal endoscopy revealed black, necrotic crusts, and two aspiration cultures confirmed *Mucor* species. Liposomal amphotericin B was started without delay; however, the disease had already extended into the orbit and frontal brain parenchyma, making surgical debridement impossible. The patient died despite aggressive antifungal therapy.

**Conclusion:**

Rhinocerebral mucormycosis should be suspected immediately in diabetic ketoacidosis patients who develop acute cranial neuropathies or sudden vision loss, even when initial neuroimaging is unrevealing. Clinical deterioration may precede radiological abnormalities in fulminant rhinocerebral mucormycosis, and delayed recognition may rapidly eliminate the possibility of surgical intervention.

## Highlights

• Mucormycosis may cause blindness before imaging changes.
• Nasal Endoscopy may enable early diagnosis.
• Delay may preclude surgery.

## Introduction

Mucormycosis is a rare but highly aggressive invasive fungal infection that is associated with substantial morbidity and mortality. Global incidence rates of mucormycosis range from 0.005 to 1.7 per million [1]. The most common genera causing mucormycosis within the order Mucorales are *Rhizopus*, *Mucor*, and *Lichtheimia*, accounting for approximately 70–80% of cases. *Rhizopus arrhizus* is the most frequently identified causative species. In contrast, *Cunninghamella, Apophysomyces, Saksenaea, Rhizomucor, Cokeromyces, Actinomucor*, and *Syncephalastrum* species alone are responsible for less than 1–5% of reported mucormycosis cases [2]. Although mucormycosis may occur in patients with haematological malignancies, organ transplantation, HIV infection, or prolonged corticosteroid exposure, poorly controlled diabetes mellitus, particularly in the presence of diabetic ketoacidosis, remains one of the strongest predisposing factors [3,4].

Rhinocerebral mucormycosis is the classic form observed in patients with diabetes [5,6]. Infection typically begins in the sinonasal mucosa and may rapidly extend to the orbit, cavernous sinus, and adjacent brain parenchyma via angioinvasion and vascular thrombosis. Early clinical manifestations may be subtle and nonspecific, such as unilateral facial swelling, headaches, nasal or sinus congestion or pain, serosanguinous nasal discharge, and fever [7]. Initial imaging findings can be non-diagnostic, which may delay recognition.

Because survival depends on prompt diagnosis, immediate antifungal therapy, and early surgical debridement, cases characterized by unusually rapid neurological or ophthalmological deterioration are of particular clinical importance. This report describes an aggressive presentation resulting in sudden bilateral vision loss within hours, despite initially normal neuroimaging.

## Case presentation

A woman in her 70 s, with a 7-year history of type 2 diabetes, presented to the emergency room with complaints of headache, fatigue, and severe hyperglycemia. Her most recent HbA1c two months earlier was 7.9%; however, she had discontinued antidiabetic therapy thereafter. She was afebrile but appeared fatigued. Initial laboratory studies revealed severe hyperglycemia (790 mg/dL), high anion-gap metabolic acidosis (pH 7.15 and bicarbonate 12 mmol/L), and ketonuria. Inflammatory markers were markedly elevated, including CRP 180 mg/L and ESR 140 mm/h, with neutrophilic leukocytosis and mildly elevated procalcitonin (0.8 ng/mL). Laboratory findings were consistent with diabetic ketoacidosis.

The patient was admitted for intravenous insulin infusion and fluid resuscitation. Empirical intravenous antibiotic therapy with ceftriaxone 2 g once daily and moxifloxacin 400 mg once daily was administered for 2 days. Because of ongoing concern for severe infection, treatment was subsequently escalated to intravenous meropenem 1 g every 8 h and intravenous teicoplanin for 5 days, given as an 800 mg loading dose followed by 400 mg once daily.

The acidosis was corrected within 24 h of hospitalization, but numbness developed on the right side of her face. Approximately 24 h later, ptosis developed in the right eye, followed within hours by complete bilateral blindness. Upon examination, both pupils were fixed and dilated with absent light reflexes, and she exhibited complete ophthalmoplegia. She was unable to voluntarily open her eyelids. Neurological examination revealed acute cranial neuropathy.

Initial non-contrast cranial CT and diffusion magnetic resonance imaging (MRI) revealed no acute abnormalities. Ophthalmology consultation revealed a pale optic disc on the right, anterior optic neuropathy, and central retinal artery occlusion; the left optic disc appeared normal. Persistent severe right frontal headache and progressive neurological deficits prompted further evaluation with contrast-enhanced cranial MRI, MR angiography, and venography, which showed bilateral frontal non-enhancing diffusion restrictions, but no vascular occlusion or cavernous sinus thrombosis. Orbital MRI demonstrated post-septal enhancing soft tissue involvement in the right orbit (Fig. 3). Paranasal sinus CT revealed diffuse mucosal thickening and soft-tissue opacification of the right maxillary, ethmoid, sphenoid, and frontal sinuses (Fig. 4).

**Fig. 1:**
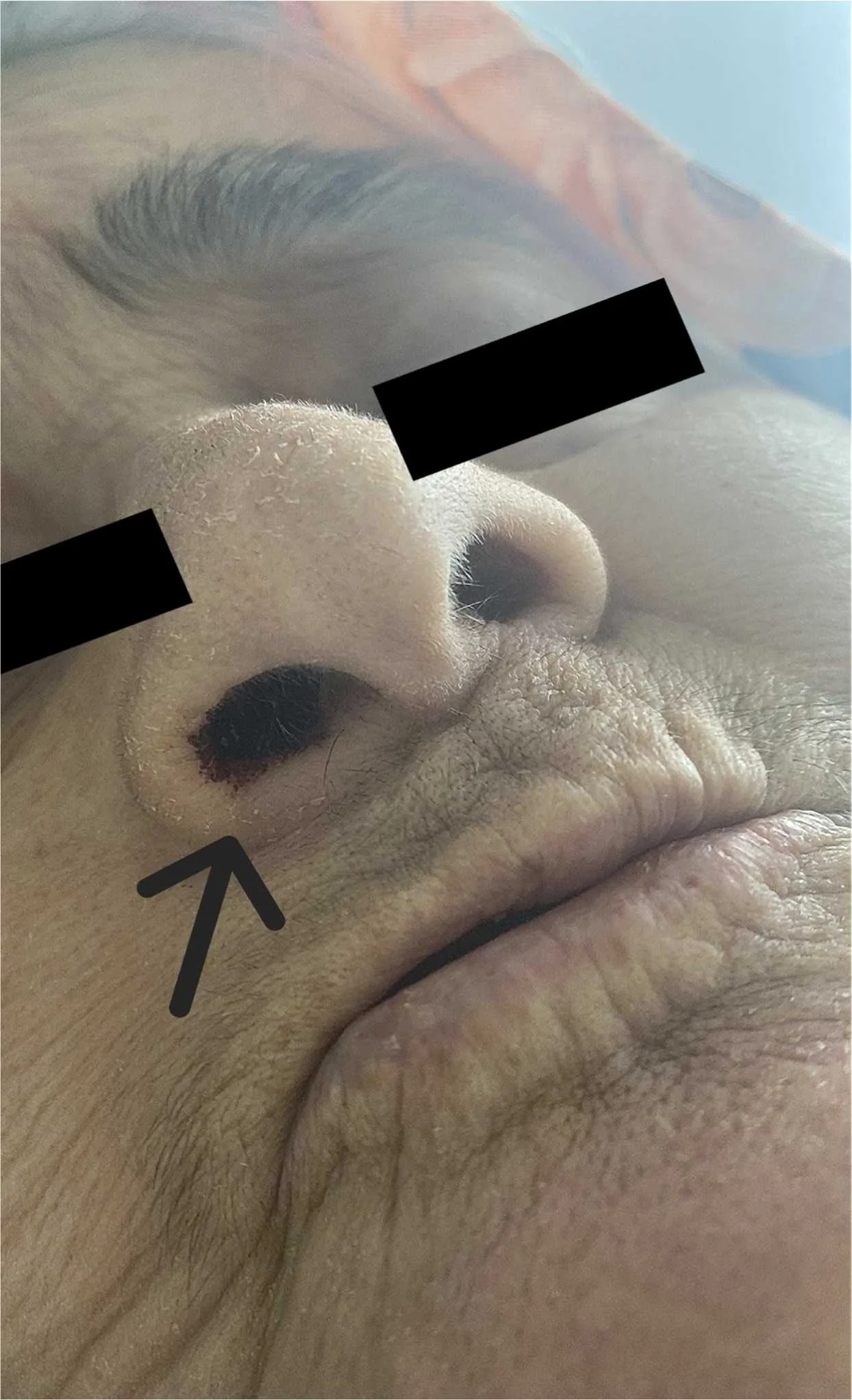
Macroscopic examination demonstrates a black necrotic eschar within the right nasal cavity.

**Fig. 2:**
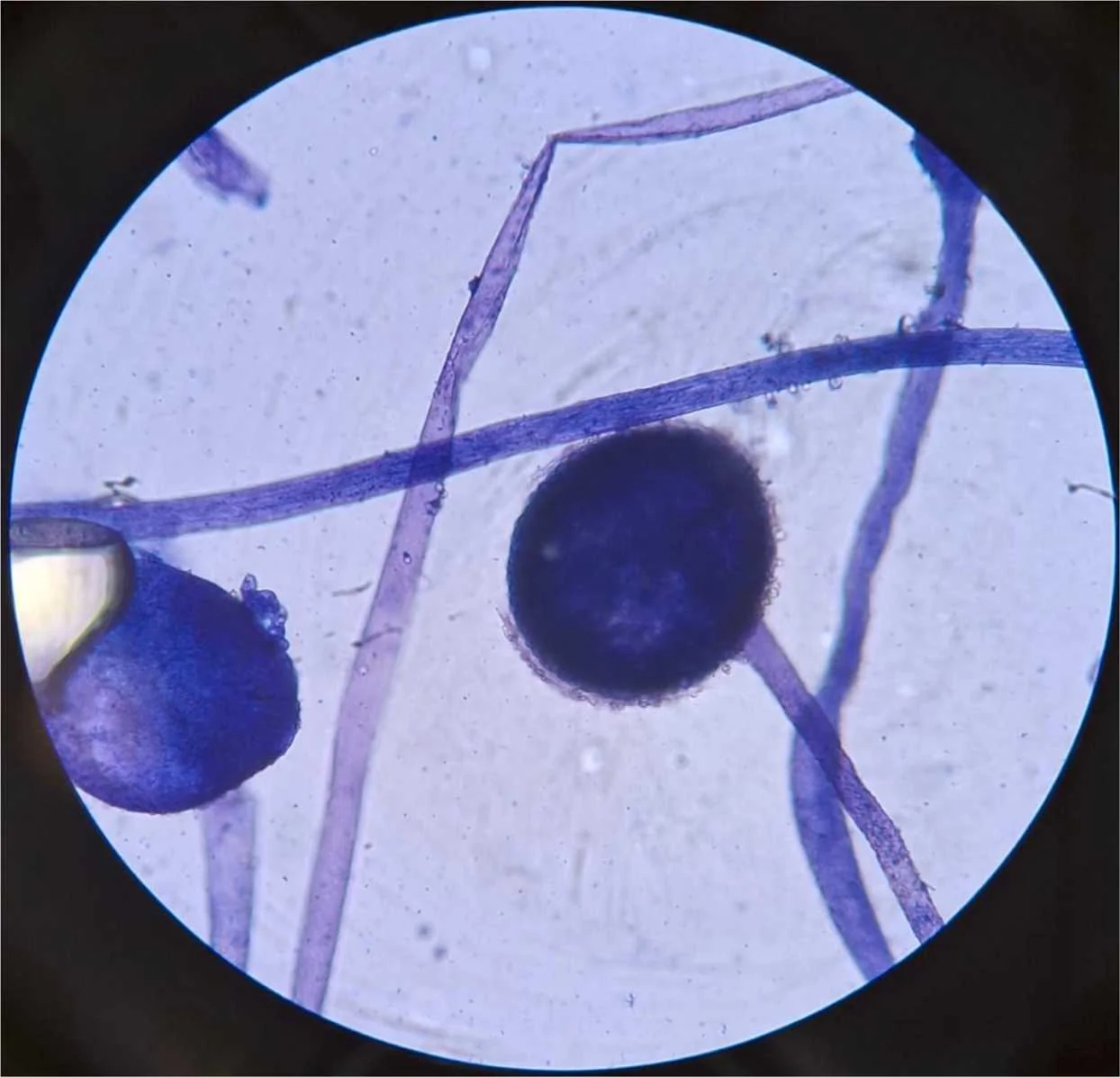
Microscopic examination of nasal aspirate demonstrating broad, ribbon-like, non-septate fungal hyphae consistent with Mucor species.

ENT consultation revealed black necrotic crusts in the right nasal cavity (Fig. 1). Nasal endoscopic biopsy could not be performed due to technical limitations; however, aspiration samples obtained twice both demonstrated growth of *Mucor* species (Fig. 2). Given the high clinical suspicion, intravenous liposomal amphotericin B (5 mg/kg/day) was initiated immediately. Despite treatment, the infection rapidly progressed with orbital and frontal lobe invasion, and the patient’s condition deteriorated. She was transferred to a tertiary centre for possible surgical debridement, but the extent of invasion rendered surgery impossible.

**Fig. 3:**
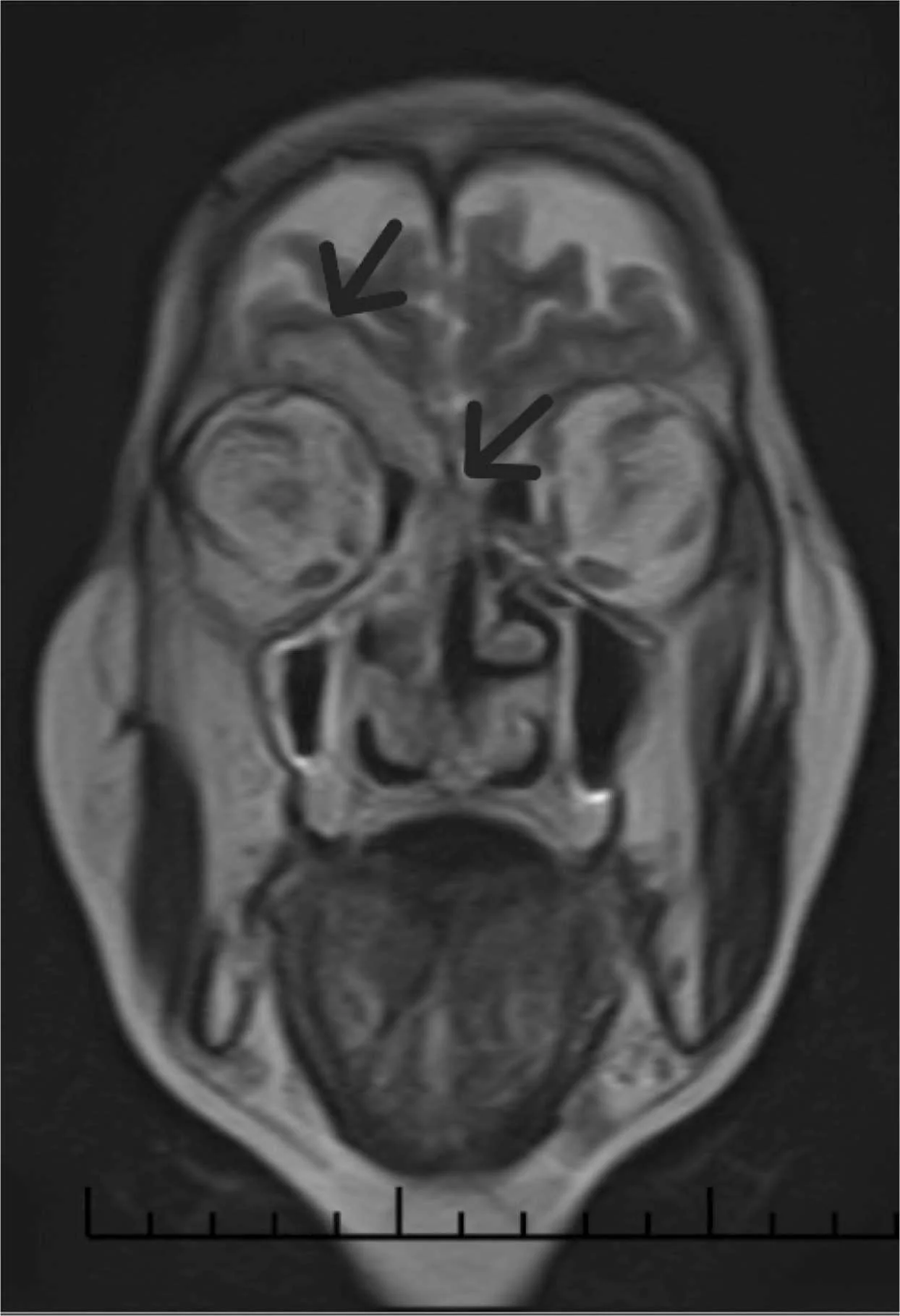
Orbital MRI demonstrating post-septal soft-tissue infiltration with intracranial extension into the right frontal lobe.

**Fig. 4:**
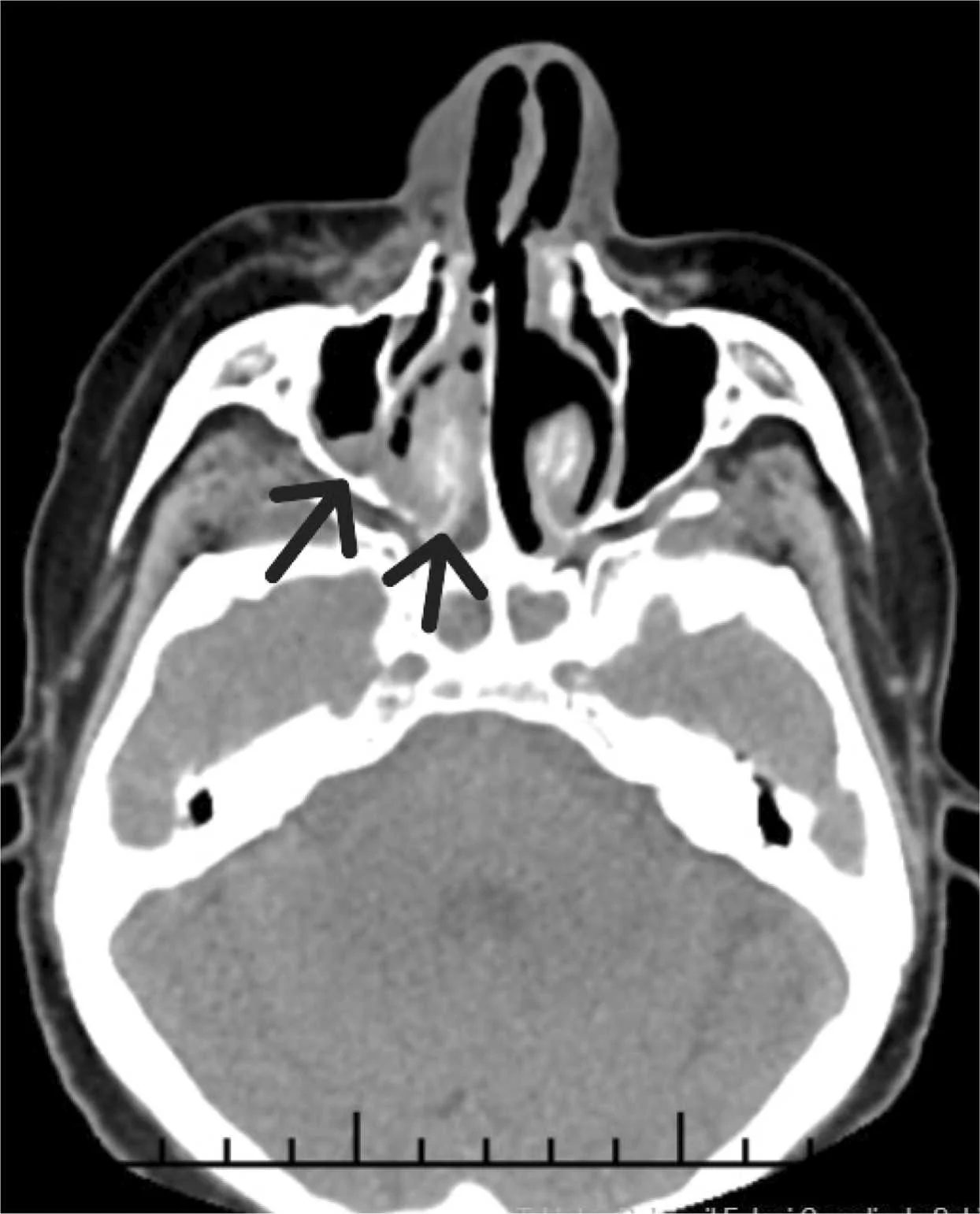
Paranasal sinus CT showing fluid collections and soft-tissue opacification within the right ethmoid and maxillary sinuses.

### Differential diagnosis

The patient's acute bilateral vision loss, ophthalmoplegia, and severe headache led to various neurological and ophthalmological diagnostic evaluations. Giant cell arteritis (GCA) was initially suspected due to the patient's age and acute vision loss. However, the absence of jaw pain, the presence of fixed dilated pupils, and complete ophthalmoplegia were atypical features for GCA. Furthermore, the normal appearance of the left optic disc was inconsistent with the bilateral arteritic anterior ischemic optic neuropathy expected in GCA. Central retinal artery occlusion was detected in the right eye, but this did not explain the rapid bilateral clinical progression. Cavernous sinus thrombosis was also considered, but ruled out due to normal MR venography and the absence of venous congestion. Orbital cellulitis and Tolosa-Hunt syndrome were also considered, but were inconsistent with the presence of rapidly evolving neurological deterioration and necrotic nasal tissue. In conclusion, the combination of cranial neuropathies, widespread sinus involvement, necrotic nasal lesions, and positive cultures confirmed the diagnosis of fulminant rhinocerebral mucormycosis.

### Treatment

The patient was treated for diabetic ketoacidosis using continuous intravenous insulin infusion, aggressive fluid replacement, and electrolyte correction. Broad-spectrum intravenous antibiotics were administered as detailed above while microbiological results were pending.

Following the development of ophthalmoplegia and rapid bilateral vision loss, further imaging was performed. However, the discovery of black necrotic tissue within the nasal cavity raised the suspicion of an invasive fungal infection. Although a nasal biopsy could not be obtained, aspiration cultures confirmed the presence of *Mucor* species.

Intravenous liposomal amphotericin B was initiated immediately at a dose of 5 mg/kg/day. The patient was urgently referred to a tertiary center for possible surgical debridement. At the tertiary center to which she was referred, a nasal biopsy was taken, and amphotericin B was increased to 10 mg/kg/day. Histopathological examination demonstrated broad, non-septate fungal hyphae with angioinvasion and scattered necrosis. Unfortunately, the extensive orbital and frontal cerebral invasion precluded surgical intervention.

### Outcome and follow-up

Despite the early initiation of liposomal amphotericin B and transfer for potential surgical management, the infection progressed rapidly. The patient’s neurological condition deteriorated, with persistent bilateral vision loss, complete ophthalmoplegia, and progressive decline in consciousness. She subsequently developed refractory metabolic and hemodynamic instability and died shortly after transfer, despite maximal supportive therapy (Table 1).

**Table 1:** Clinical timeline of disease progression.

**Time from Admission**	**Clinical Event**
Admission	Presentation with headache, fatigue, severe hyperglycemia, and diabetic ketoacidosis
Within 24 h	Development of right-sided facial numbness
Approximately 48 h after admission	Ptosis followed by sudden complete bilateral vision loss
Initial imaging	Cranial CT and diffusion MRI were unremarkable
Subsequent evaluation	Progressive ophthalmoplegia and elevated inflammatory markers prompted further imaging
Repeat imaging	Orbital MRI showed post-septal orbital involvement; paranasal sinus CT revealed extensive pansinusitis
ENT examination	Black necrotic crusts were identified in the right nasal cavity
Microbiology	Two aspiration cultures confirmed Mucor species
Treatment	Immediate initiation of liposomal amphotericin B
Clinical course	Rapid orbital and frontal lobe invasion despite antifungal therapy
Outcome	Surgical debridement deemed impossible; patient died despite aggressive treatment

## Discussion

Rhinocerebral mucormycosis is one of the most fulminant forms of invasive fungal infection and is strongly associated with uncontrolled diabetes, particularly diabetic ketoacidosis. Acidosis enhances fungal proliferation by impairing neutrophil function and increasing the available free iron, facilitating rapid angioinvasion and subsequent tissue necrosis [[3], [4], [5]]. A retrospective review of this patient revealed poor glycemic control and recent discontinuation of insulin and oral antidiabetics, creating an optimal environment for rapid fungal dissemination.

Early manifestations of rhinocerebral mucormycosis, such as headache, nasal congestion, facial pain, or serosanguineous discharge, may be subtle and nonspecific. As the infection progresses, cranial neuropathies, ptosis, ophthalmoplegia, and sudden vision loss can develop due to vascular invasion and involvement of the orbital apex and cavernous sinus [5,8]. In our case, the patient had no fever or obvious facial edema, and the initial CT and MRI findings were unremarkable despite rapidly evolving neurological deficits.

The most striking feature of this case was the discrepancy between the rapidly progressive neurological deterioration and the initially unremarkable neuroimaging findings. This discrepancy may delay diagnosis and life-saving antifungal treatment. To our knowledge, only a few reports have described fulminant bilateral blindness occurring before radiologically detectable abnormalities in rhinocerebral mucormycosis Table 2 [7,8]. Clinicians should be aware that in rhinocerebral mucormycosis, angioinvasive vascular and neural damage may precede detectable structural abnormalities in early imaging studies. Reasons why cranial imaging may initially appear normal include the infection presenting as nonspecific sinusitis [3], progressing with angioinvasion and perineural involvement before bone destruction [4], and the inability to visualize microthrombi and tissue necrosis in the early stages [9]. Furthermore, because the disease can progress rapidly, an initially normal imaging result can transform into a devastating, extra-sinus (orbital or intracranial) disease within days. In our case, there were two days between the patient's normal cranial CT-MRI results at presentation and the subsequent radiological abnormalities.

**Table 2:** Clinical presentations, initial imaging findings, and outcomes of previously reported rhinocerebral mucormycosis cases.

**Author**	**Initial Presentation**	**DM/DKA**	**Clinical Progression**	**Initial Imaging Findings**	**Surgery**	**Outcome**
Erdil et al. [10]	Eye pain and swelling	DM	Vision loss	Orbital cellulitis and cerebellar diffusion restriction	Debridement	Death
Umemura [11]	Diplopia and headache	DM	Total left ophthalmoplegia and visual loss	Orbital apex mass lesion	Major surgery	Death
Galleta et al. [12]	Sinus pain, bilateral periorbital edema	DM	Bilateral ophthalmoplegia	Bilateral cavernous sinus syndrome	Endoscopic sinus surgery	Death
Mulki et al. [13]	Headache, mental status change	DKA	Bilateral ophthalmoplegia, vision loss	Mucosal thickening in the ethmoid sinus	Debridement	Recovery
Aicher et al. [14]	Frontal headache, ptosis	DM	Cavernous sinus thrombosis and periorbital cellulitis	Optic neuritis and minimal edema of the eye muscles	Partial surgical resection	Death
Diongue et al. [15]	Eye pain and swelling	DKA	Loss of consciousness, spread of necrosis in the parietal-temporal region.	Chronic pansinusitis, and diffuse cerebral hypodensity	Could not be performed	Death
Our case	Headache, facial numbness	DKA	Bilateral blindness	Normal CT and DWI MRI	Could not be performed	Death

Our case also highlights that rapidly progressive cranial neuropathies in diabetic ketoacidosis require urgent sinonasal evaluation, even in the absence of definitive early imaging abnormalities. Therefore, nasal endoscopy is essential, as black necrotic crusts on the nasal mucosa or palate may be the earliest clinical clue, even when external lesions are absent. In this patient, the identification of black necrotic tissue prompted urgent antifungal therapy, and nasal aspiration cultures subsequently confirmed the presence of Mucor species. Bilateral acute vision loss initially raised suspicion for giant cell arteritis; however, fixed dilated pupils, complete ophthalmoplegia, and the pattern of optic neuropathy were inconsistent with this diagnosis.

The management of mucormycosis requires immediate initiation of high-dose liposomal amphotericin B and prompt surgical debridement. Delays of even a few hours may allow for catastrophic angioinvasion. Unfortunately, extensive orbital and frontal lobe invasion in this patient precluded surgical intervention, which is a factor strongly associated with mortality [6]. Despite aggressive antifungal therapy, the patient’s condition rapidly deteriorated.

## Conclusion

This case underscores how mucormycosis can progress with catastrophic rapidity in diabetic patients and highlights the need for a high index of suspicion when acute cranial neuropathies or sudden vision loss occur—even when early imaging appears normal. Early nasal endoscopy and immediate therapy are critical for improving patient survival.

## CRediT authorship contribution statement

**Nur Beyza Tukek Kilicaslan:** Writing – review & editing, Writing – original draft, Visualization, Validation, Supervision, Software, Project administration, Methodology, Investigation, Funding acquisition, Formal analysis, Data curation, Conceptualization. **Muhammed Enes Kardan:** Writing – review & editing, Supervision. **Hulya Duran:** Writing – review & editing, Supervision.

## Ethics declaration

Written informed consent to take part in the study and to publish the article has been obtained from all participants or their legal representatives. The privacy rights of participants have been observed.

This study was performed in compliance with relevant laws, regulatory frameworks and guidelines where the research took place. Ethics committee approval was not required under relevant laws and institutional guidelines. Since it is a case report and not a clinical trial, it does not require review by an ethics committee.

This research follows the CARE guidelines and the CARE checklist.

## Patient consent

Written informed consent has been obtained from the patient's first-degree relative to publish this paper because the patient has died.

## Declaration of Competing Interest

The authors declare that they have no known competing financial interests or personal relationships that could have appeared to influence the work reported in this paper.
